# Dynamics of Genotypic Mutations of the Hepatitis B Virus Associated With Long-Term Entecavir Treatment Determined With Ultradeep Pyrosequencing: A Retrospective Observational Study

**DOI:** 10.1097/MD.0000000000002614

**Published:** 2016-01-29

**Authors:** Xia-Xia Zhang, Min-Ran Li, Ying Cao, Ren-Wen Zhang, Yu Zhang, Fang Li, Hong-Li Xi, Xiao-Yuan Xu

**Affiliations:** From the Department of Infectious Disease, Peking University First Hospital, No.8 Xishiku Street, Xicheng District, Beijing, China.

## Abstract

The aim of the study is to explore the evolution of genotypic mutations within the reverse transcriptase region in partial virological responders (PVRs) receiving long-term entecavir (ETV) treatment.

A total of 32 patients were classified as completely virological responders (CVRs) (n = 12) or PVRs (n = 20). Five partial responders were hepatitis B virus (HBV)-DNA positive after long-term therapy, which lasted for >3 years. A total of 71 serum samples from these 32 patients were assayed by ultra-deep pyrosequencing (UDPS): 32 samples were from all patients at baseline, and 39 were from PVRs with sequential inter-treatment.

Approximately 84,708 sequences were generated per sample. At baseline, the quasispecies heterogeneity did not significantly differ between the 2 groups. The frequencies of substitutions indicating pre-existence of nucleos(t)ide analog resistant (NAr) mutants ranged from 0.10% to 6.70%, which did not statistically differ between groups either. However, the substitutions associated with the NAr mutants were significantly different from those associated with the non-NAr mutants in 13 patients; 6 of these patients were PVRs and the others were CVRs. Five patients were HBV DNA positive after regular ETV monotherapy for >3 years, and 4 of these patients underwent mild NAr substitution fluctuations (<20%). One patient developed virological breakthrough while bearing single, double, and triple (rtL180 M, rtM204 V, rtS202G) substitutions. In addition to the common substitutions, unknown amino acid substitutions, such as rtL145 M/S, rtF151Y/L, rtR153Q, rtI224 V, rtN248H, rtS223A, rtS256C, need to be further verified.

NAr substitutions are observed at frequencies of 0.10% to 6.7% before therapy. Long-term ETV therapy generally results in virological responses, as long as the proportion of resistance mutations remains at a relatively low level. Genotypic resistance to ETV is detected in all PVRs receiving long-term ETV therapy.

## INTRODUCTION

Hepatitis B virus (HBV) is a global disease that chronically infects ∼350 million people worldwide and that plays a vital role in liver-related cirrhosis and hepatocellular carcinoma.^1–4^ In addition to interferon, 5 oral antiviral drugs have been approved for HBV therapy to suppress HBV virological activities, including nucleosides (lamivudine, telbivudine, entecavir) and nucleotides (adefovir, tenofovir), which directly inhibit the HBV reverse transcriptase (RT) enzyme and effectively suppress viral replication.^5^ Entecavir (ETV), as the most potent antiviral drug, is the first-line antiviral drug for naive patients and can successfully suppress viral replication, with a high barrier to resistance, to avoid disease progression.^6^ However, long-term use of nucleos(t)ide analog (NA) can cause the emergence of drug resistance or cross-resistance, which is the main obstacle for antiviral therapy success. The lacking of viral-encoded RNA-dependent DNA polymerase proofreading activity and the extremely high rate of viral replication lead to the generation of mutations at nucleotide position within the genome in HBV virus.^7^ Several reports based on less potent NAs have demonstrated that these analogs may cause the accumulation of related resistant variants or pre-existing variants, which are defined by the presence of minor drug-resistance mutations in treatment-naive hepatitis patients.^8,9^

Clinical resistance which based on amino acid substitutions resulting from genotypic mutations causes viral population fluctuations.^10,11^ These fluctuations in viral populations can be explained by quasispecies (QS) dynamics (a series of highly correlated, but not identical, dynamic populations composed of variants and reorganizations of the genome that are influenced by genetic variation, competition, and choice selection), which has been reported by many studies.^12,13^ QS include a series of mutants at different fitness levels, and predominant mutants with greater fitnesses may cause virological and biochemical breakthroughs, resulting in disease process acceleration.^14^ HBV RT QS can be used to predict the clinical curative effects of the virological response during the early stage of therapy.^15,16^ Therefore, it is important to discover the link between dominant substitution populations of HBV and the sensitivities of antiviral drugs to reasonably guide the treatment.

Early HBV responses can guide the therapeutic regimens for primary nonresponders and partial responders based on less potent NAs, such as lamivudine and telbivudine, with less data regarding ETV or tenofovir.^17,18^ Reports have specially demonstrated that long-term ETV treatment could ultimately result in a virological response in primary nonresponders.^19,20^

Considering the above situations, the present study aimed to explore the specific kinetics of genotypic mutations in partial virological responders (PVRs) receiving long-term ETV and to analyze the relationships between HBV RT QS and clinical curative effects in completely virological responders (CVRs) and PVRs.

Some common techniques, such as direct sequencing and the line probe assay, were used in present research works regarding HBV drug-resistance mutations and QS dynamics. However, these techniques can only be used to detect the frequency of at least 5% of the HBV QS and can be used to identify only previously known substitutions.^21^ Currently, cloning is prevalent in known substitutions identification but cumbersome and time-consuming, and was not able to identify random minor variants.^22^ Now, next-generation sequencing techniques become the optimal methods to overcome the above disadvantages for rare mutations exploration.

## PATIENTS AND METHODS

### Study Subjects

A total of 61 patients were collected from September 2006 to December 2007 from the Department of Infectious Disease of Peking University First Hospital (China). All patients met the clinical diagnostic criteria for chronic hepatitis B (CHB). The patients regularly received ETV (0.5 mg, qd) for at least 7 years. The HBV DNA was undetectable after treatment for 48 weeks, which was defined as virological response, whereas being a PVR was being HBV DNA positive after 48 weeks, but with a decrease of >1 log10 copy/mL compared with baseline. Virological breakthrough was defined as serum HBV DNA increase of >1 log10 IU/mL compared with the nadir level. We analyzed the baseline of the CVRs and observed the continuous dynamics of the PVRs, particularly among those patients who were HBV DNA positive for >3 years. The inclusion criteria were (a) patients who were naive to NA treatment and (b) patients who had been receiving monotherapy with ETV for at least 7 years, without termination. The exclusion criteria were (a) serious liver-related complications (decompensated liver cirrhosis, hepatocellular carcinoma, liver transplantation), (b) coinfection with human immunodeficiency virus (HIV) or hepatitis C virus (HCV), (c) combination therapy with other NAs, (d) a lack of comprehensive samples at testing time points, and (e) poor compliance. A final total of 32 treatment-naive patients were included in the study, and a total of 71 serum samples from these 32 patients were assayed by ultradeep pyrosequencing (UDPS): 32 samples were from all patients at baseline, and 39 were from PVRs with sequential intertreatment.

### Ethics Statement

The study was in compliance with the Helsinki Declaration and was approved by the Medical Ethics Committee of Peking University First Hospital. All the participants gave the written informed consent.

### Test for Related Virological Indicators

Liver biochemistries and serum HBV DNA levels were tested a lot of points (baseline and 0.5, 1, 2, 3, 4, 5, 6, and 7 years) after ETV therapy. The biochemical indicators (alanine aminotransferase, ALT, and aspartate transaminase, AST) were tested using an automatic biochemical analyzer.^23^ HBV DNA was quantified using a COBAS TaqMan assay (Roche Diagnostics, Basel, Switzerland), and the lowest limit of detection was 20 IU/mL. Other serological markers (HBsAg, anti-HBsAg, HBeAg, anti-HBe, anti-HBc) were measured using ELISA kits (Abbott Laboratories, Chicago, IL). HBV genotypes were determined by comparing the generated preS/S gene sequences with GenBank (NCBI) data.^24^

### PCR Amplification and UDPS Data

HBV DNA was extracted from 1 mL of available serum samples according to the manufacturer's instructions (QIAamp UltraSens Virus kit, Qiagen, Germany). A 913-bp fragment of the HBV reverse transcriptase (nt84 to 997) was first PCR-amplified with primers HBVRTfw1: 5′-GGCTCCAGTTCAGGAACAGT-3′ and HBVRTrv1: 5′-GCAAAGCCCAAAAGACCCACAAT-3′. The second 383-bp PCR fragment (nt 515 to 898) was amplified with primers HBVRTfw2: 5′-CTACCAGCACGGGACCAT-3′ and HBVRTrv2: 5′-TCCTGTGGTAAAGTACCCCA-3′. The conditions for PCR were 40 cycles of 98°C for 20 s, 60°C for 30 s, and 72°C for 5 min. PCR products were analyzed via electrophoresis through 1% agarose gel and ethidium bromide staining. Then the PCR amplicons were purified with the QIA quick PCR purification kit (Qiagen) and quantified with an Agilent 2100 bioanalyzer (Agilent Life Science, Santa Clara, CA). Afterwards, the amplicons were sequenced on MiSeq platform (Illumina, American).

For the sake of high sequencing error rates and homopolymeric bias, all UDPS reads were subject to additional error correction by the means of Sanger method (Applied Biosystems, Foster City, CA). All reads from UDPS and Sanger methods were aligned to homologous sequences from the GenBank database. Meanwhile, we figured out the error rates for homopolymeric (0.0025) and nonhomopolymeric regions (0.0011) separately. The cutoff value was defined as 1%. In UDPS, every sample had a Barcode tag in order to identify the sample from a specific patient. The paired-end (PE) reads were combined by FLASH (v1.2.7), and quality controlled by FastQC. The combined reads which contains >20% low-quality bases (quality score < 20) or has >5 extra low-quality bases (quality score <5) was discarded. The reads numbers of obtained libraries were varied from 50,319 to 176,806, with a mean value of 84,708 reads per sample. Reads aligned with reference sequences by threshold of ≥90% coverage were reserved for further analysis.

### Sequence Analysis

All gap regions were filtered by in-house script by Clustal X (version 1.8) and NCBI Blast. QS complexity and diversity were measured based on Shannon entropy and other parameters, such as Hamming distance, the synonymous substitutions per synonymous site (dS) and the nonsynonymous substitutions per nonsynonymous site (dN), which were calculated by the MEGA 5 model.^15,25^

### Statistical Analysis

All data were performed with the SPSS 17.0 (SPSS Inc, Chicago, IL). Continuous variables were expressed as mean ± SD or median and range. We compared the characteristics and QS between the CVRs and the PVRs using Student's *t* test and Fisher's exact test. The differences between the resistance and nonresistance mutations of each patient were assessed using the Mann–Whitney *U* test. *P* values of < 0.05 was considered statistically significant.

## RESULTS

### Patients and Samples

At last, 32 consecutive CHB patients were included in the study, and they were classified as CVRs (n = 12) or PVRs (n = 20), as assessed based on virus load after treatment for 48 weeks. The clinical characteristics of all patients are listed in Table 1. At baseline, the DNA levels were significantly different (6.37 ± 1.56 log_10_ IU/mL vs 7.71 ± 0.77 log_10_ IU/mL, *P* = 0.016), whereas other factors, such as gender, age, HBeAg status, ALT levels, HBsAg levels, and genotype, were not significantly different. Four patients (No. 1–4) with long-term ETV monotherapy presented detectable HBV DNA levels for > 3 years, and 1 (No. 5) patient underwent virological breakthrough.

**TABLE 1:**
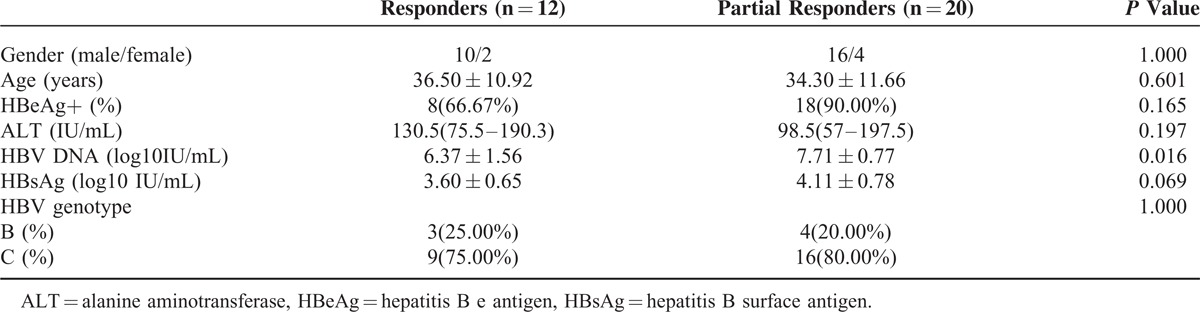
Clinical Characteristics of the 32 Treatment-Naive Chronic Hepatitis B Patients

### HBV QS Change at Baseline

Table 2 shows that the QS complexity and diversity were not significantly different between the CVRs and the PVRs at baseline (*P *> 0.05).

**TABLE 2:**
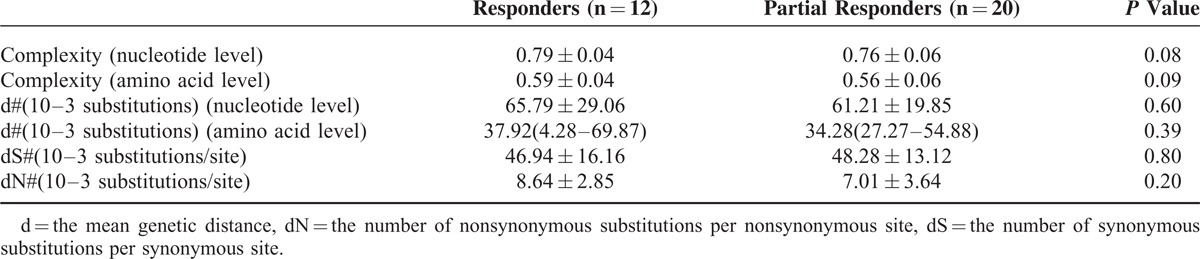
Quasispecies Complexity and Diversity at Baseline

### NA-Related Resistance Mutations at Baseline

Table 3 lists the frequencies of NAr substitutions in all CHB patients before therapy, as determined by UDPS. In the PVR group, all treatment-naive patients harbored rtA181 V/T substitutions (ranging from 1.1% to 3.8%) and rtN236T substitutions (ranging from 1.5% to 6.1%). Fourteen patients harbored rtM204I/V substitutions of at least 1% (ranging from 1% to 3.5%), and 5 of these patients also presented rtL180 M substitutions (ranging from 1.8% to 2.6%) and rtS202G substitutions (ranging from 1% to 3.4%), which are both known to confer resistance to ETV. Additionally, 2 patients harbored rtV214A substitutions (1% and 1.1%). Other substitutions (rtI169T/V, rtV173A/M, rtT184A/I, rtA194 V/T, rtQ215R/H, rtM250 V) were presented at low levels (<1%). In the CVR group, all treatment-naive patients harbored rtA181 V/T substitutions (ranging from 1.2% to 4.6%). Eight patients harbored rtM204I/V substitutions of at least 1% (ranging from 1% to 3.6%); 2 of these patients also presented rtL180 M substitutions (2.3% and 2.4%) and rtS202G substitutions (3.2% and 3.5%), whereas the other 6 harbored a low frequency of rtS202G substitutions (ranging from 1% to 1.4%), with 1 harboring rtQ215R/H substitutions (6.7%) and another harboring rtV173A/M substitutions (1.7%). Other substitutions (rtI169T/V, rtT184A/I, rtA194 V/T, rtV214A, rtM250 V) were present at low levels (<1%).

**TABLE 3:**
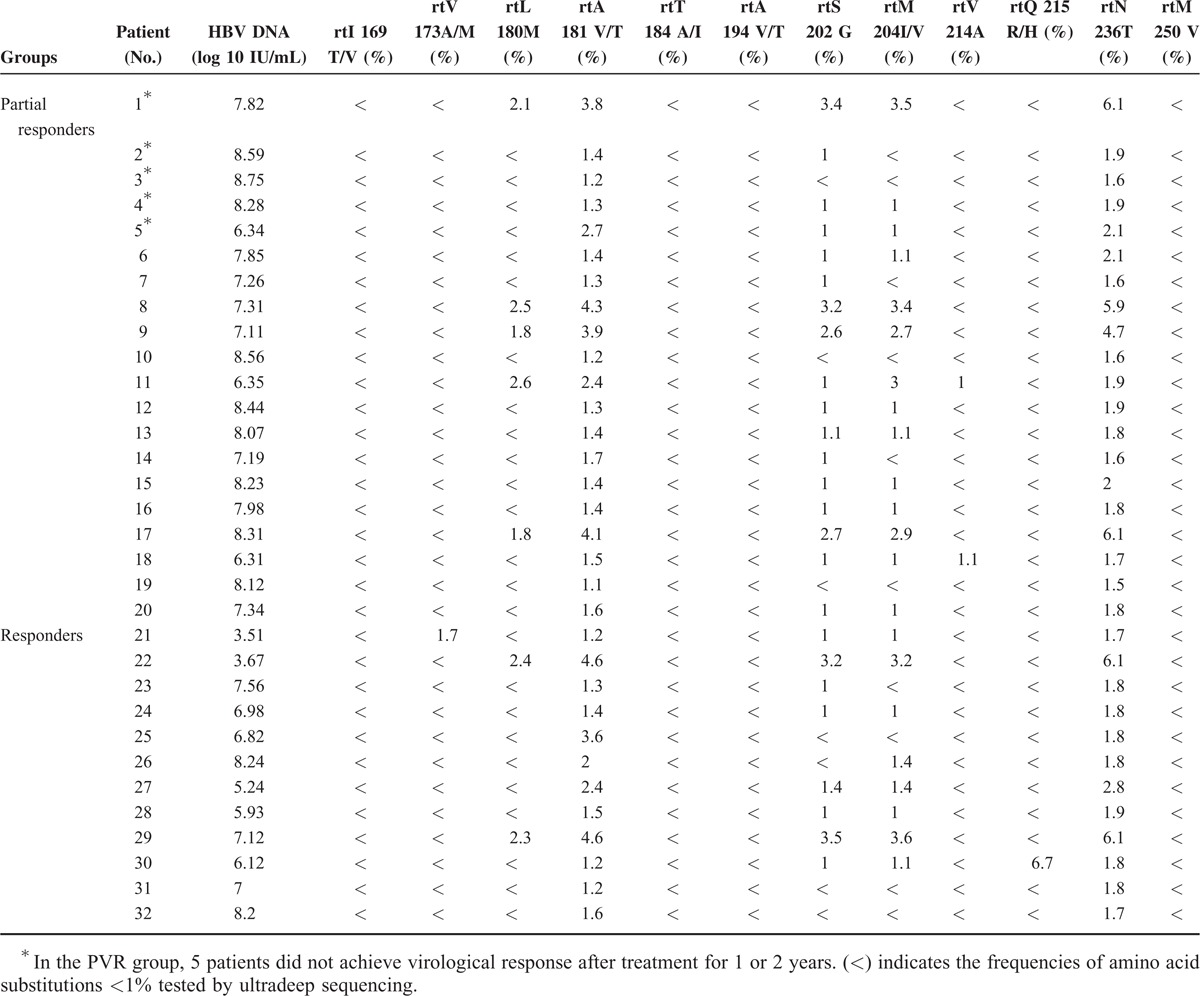
Frequencies of Amino Acid Substitutions in the 32 Treatment-Naive Chronic Hepatitis B Patients

NAr mutations did not significantly differ between the 2 groups, and when we further analyzed the resistance and nonresistance mutations, we observed that 13 patients displayed significant differences, including 6 patients from the PVR group and 7 patients from the other group (Table 4). Among the 20 patients in the PVR group, 15 achieved a virological response after treatment for 1 or 2 years. Moreover, 2 patients had undetectable HBV DNA levels until 3 years, 2 patients had undetectable HBV DNA until 5 years, and 1 experienced virological breakthrough. The dynamic QS changes in those 5 patients were further studied to explore the mechanisms underlying their responses.

**TABLE 4:**
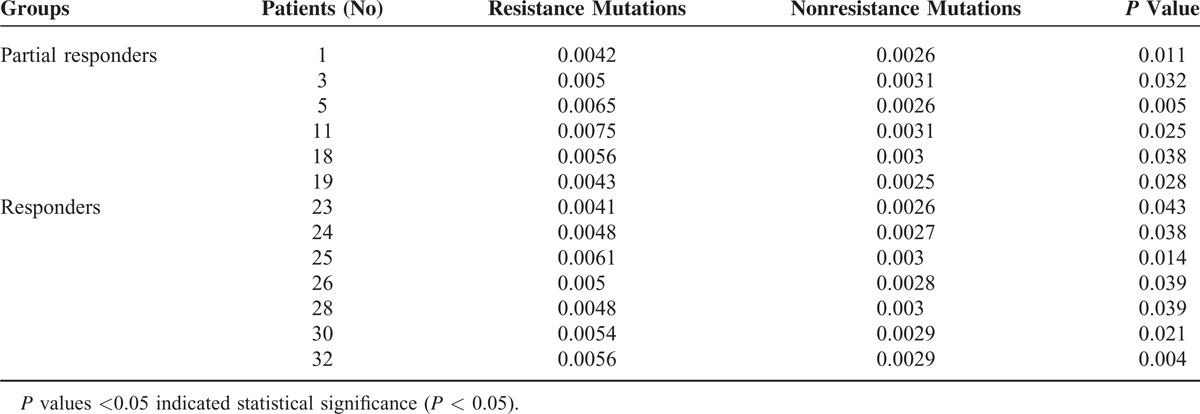
Comparison of Resistance and Nonresistance Mutations for HBV Patients at Baseline

**FIGURE 1:**
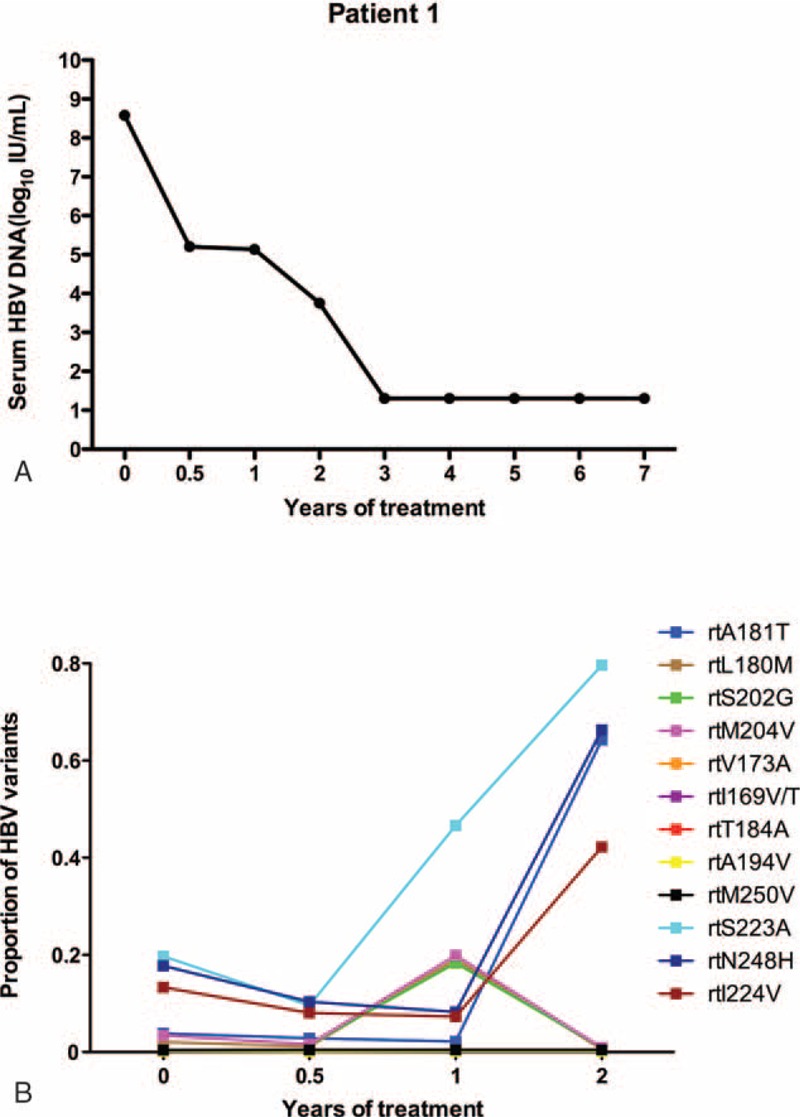
The dynamic changes in HBV DNA levels and resistant variants of the RT domains during ETV treatment of patient 1. (A) The serum HBV DNA level declined gradually and was undetectable after treatment for 3 years and followed-up for 7 years. (B) The dynamic changes in the RT domains of HBV variants, as determined by UDPS. At baseline, this patient displayed rtM204I/V (3.5%), rtL180 M (2.1%), and rtS202G (3.4%) substitutions. A wave of resistant variants (rtM204 V [19.95%], rtL180 M [19.12%], and rtS202G [18.33%]) was detected at 1 year and then decreased to <1% by 2 years. The rtA181T substitution rose from 2.17% to 64.15% by 2 years and certain nonresistance mutations (rtN248A, rtI224 V, and rtS223A) increased significantly. ETV = entecavir, DNA = di-ribonucleic acid, HBV = hepatitis B virus, RT = reverse transcriptase, UDPS = ultra-deep pyrosequencing.

Patient 1 responded suboptimally to ETV: the serum DNA level declined gradually, and HBV DNA was only undetectable after 3 years of treatment (Figure 1A). Figure 1B indicates the dynamic changes in the RT domains of HBV variants, as determined by UDPS. At baseline, this patient displayed rtM204I/V (3.5%), rtL180 M (2.1%), and rtS202G (3.4%) substitutions, and the resistance and nonresistance mutations frequencies were significantly different (*P* = 0.011). The wave of resistant variants detected at 1 year, including several single amino acid substitutions, such as rtM204 V (19.95%), rtL180 M (19.12%), and rtS202G (18.33%), decreased to <1% by 2 years. Meanwhile, the rtA181T substitution rose from 2.17% to 64.15% by 2 years, and certain nonresistance mutations (rtN248A, rtI224 V, and rtS223A) had increased significantly.

**FIGURE 2:**
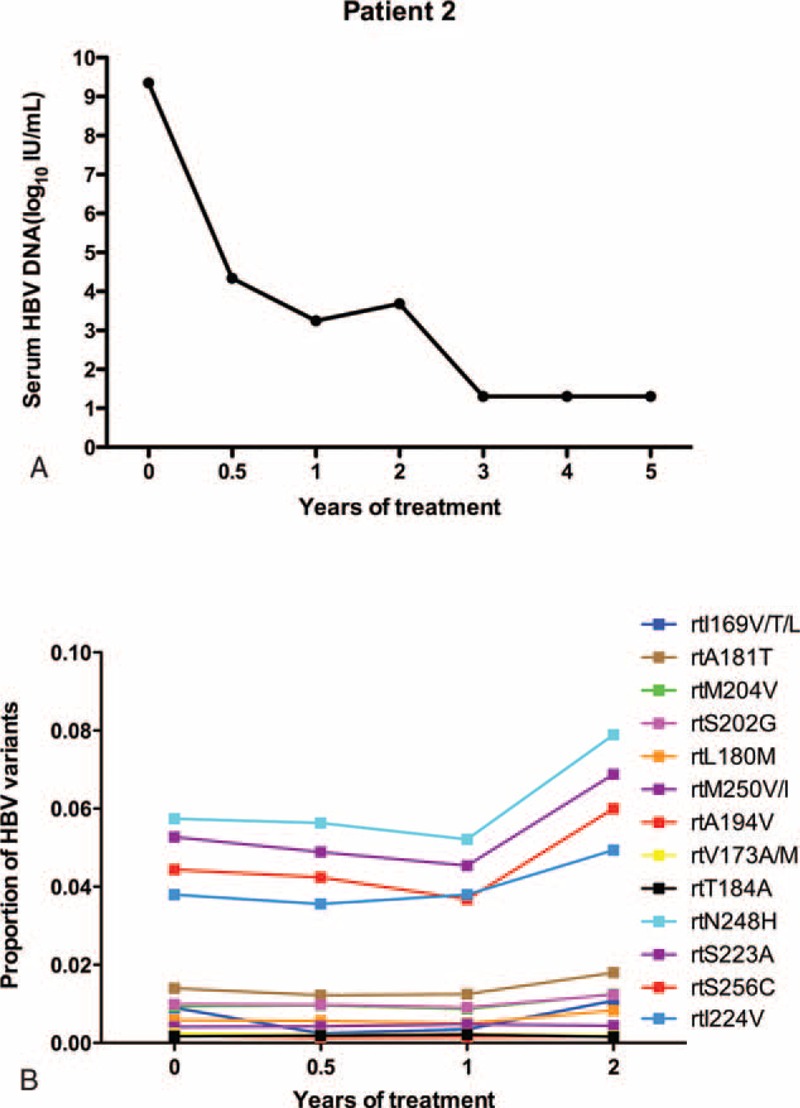
The dynamic changes in HBV DNA levels and resistant variants of the RT domains during ETV treatment of patient 2. (A) The serum HBV DNA level was undetectable after treatment with ETV for 3 years. (B) At baseline, the virus harbored an rtA181T substitution (1.4%), and other resistance mutations were present at low levels (<1%). These mutations did not obviously fluctuate during the follow-up period. Several other variants fluctuated at relatively high levels (ranging from 3.56% to 7.89%), including rtN248H, rtS223A, rtS256C, and rtI224 V mutations. ETV = entecavir, DNA = di-ribonucleic acid, HBV = hepatitis B virus, RT = reverse transcriptase.

As in patient 1, the serum HBV DNA level of patient 2 was undetectable after treatment with ETV for 3 years (Figure 2A). At baseline, patient 2 harbored an rtA181T substitution (1.4%), and other resistance mutations were present at low levels (<1%). These mutations did not obviously fluctuate during the follow-up period. However, several other variants fluctuated at relatively high levels (ranging from 3.56% to 7.89%), including rtN248H, rtS223A, rtS256C, and rtI224 V (Figure 2B).

**FIGURE 3:**
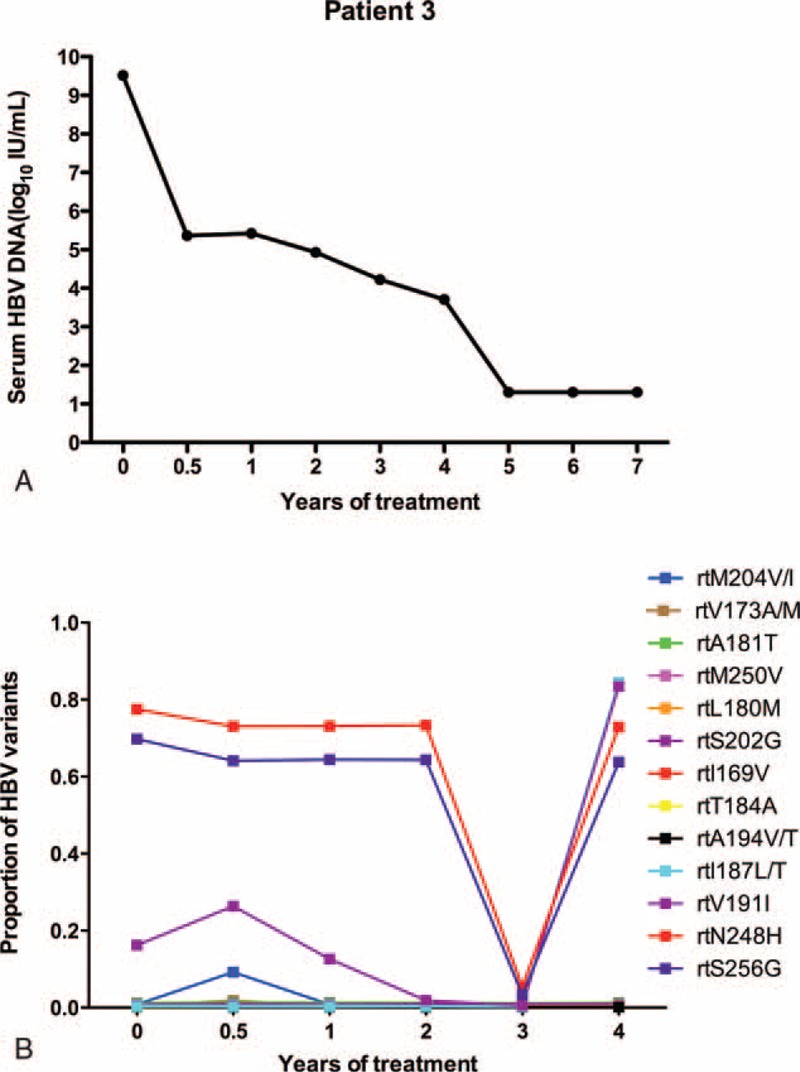
The dynamic changes in HBV DNA levels and resistant variants of the RT domains during ETV treatment of patient 3. (A) The serum HBV DNA level declined gradually and was undetectable by the 5th year of ETV treatment. (B) At baseline, the virus only harbored an rtA181T substitution (1.2%) and had low fluctuations during the follow-up period. Resistant variants (rtM204I/rtV173 M/A) increased to 9.23%/1.87% in 24 weeks and declined to <1% at 1 year. Two variants (rtI187L/rtV191I) rose to a high level during the 4th year (84.43% /83.41%), and 2 other variants, namely, rtN248H and rtS256G, were maintained at a high level (>60%). ETV = entecavir, DNA = di-ribonucleic acid, HBV = hepatitis B virus, RT = reverse transcriptase.

Patient 3 responded suboptimally to ETV: the serum DNA level declined gradually and was only undetectable by the 5th year of treatment (Figure 3A). At baseline, the virus only harbored an rtA181T substitution (1.2%) and had low fluctuations during the follow-up period. Resistant variants (rtM204I/rtV173 M/A) increased to 9.23%/1.87% in 24 weeks and declined to <1% at 1 year. In addition, 2 variants (rtI187L/rtV191I) rose to a high level during the 4th year (84.43%/83.41%), and 2 other variants, namely rtN248H and rtS256G, were maintained at a high level (>60%) (Figure 3B).

**FIGURE 4:**
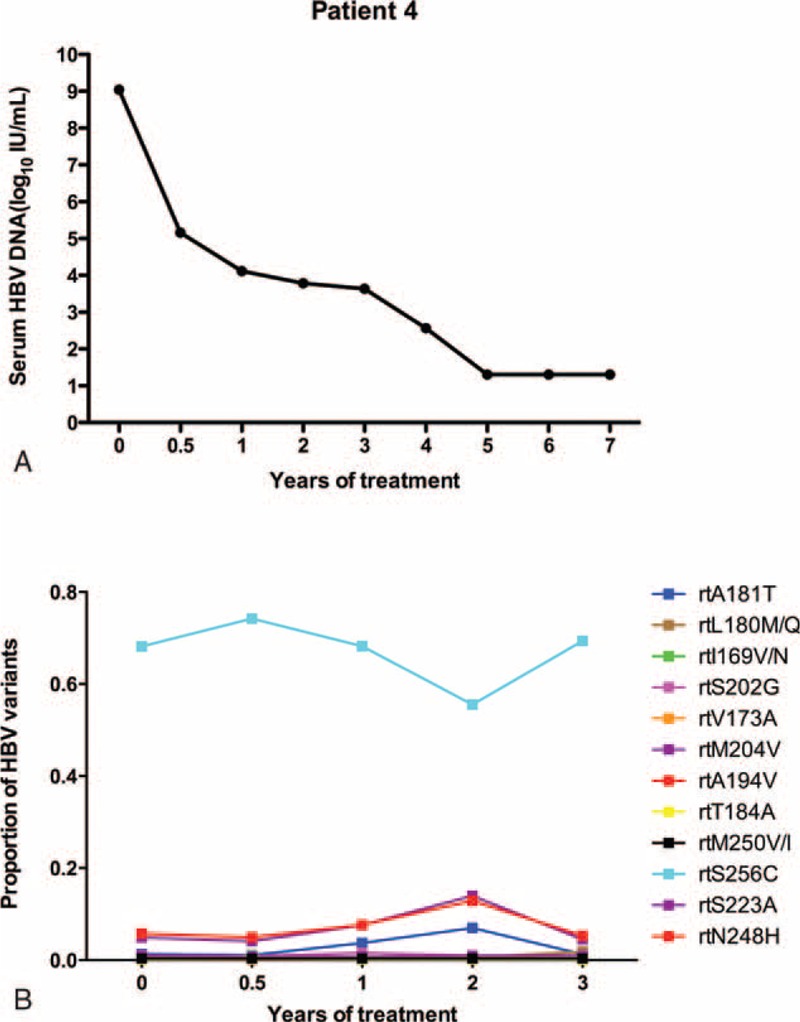
The dynamic changes in HBV DNA levels and resistant variants of the RT domains during ETV treatment of patient 4. (A) The serum HBV DNA level was undetectable after treatment for 5 years. (B) Resistance mutations displayed low levels of fluctuation (<7%), and nonresistant variants (rtN248H/rtS223A) rose to 12.88%/13.1% by 2 years. Additionally, rtS256G substitutions were maintained at a high level (>55%).ETV = entecavir, DNA = di-ribonucleic acid, HBV = hepatitis B virus, RT = reverse transcriptase.

As in patient 3, the serum HBV DNA of patient 4 was undetectable after treatment for 5 years (Figure 4A). Resistance mutations displayed low levels of fluctuation (<7%), and other nonresistant variants (rtN248H/rtS223A) rose to 12.88%/13.1% by 2 years, whereas rtS256G substitutions were maintained at a high level (>55%) (Figure 4B).

**FIGURE 5:**
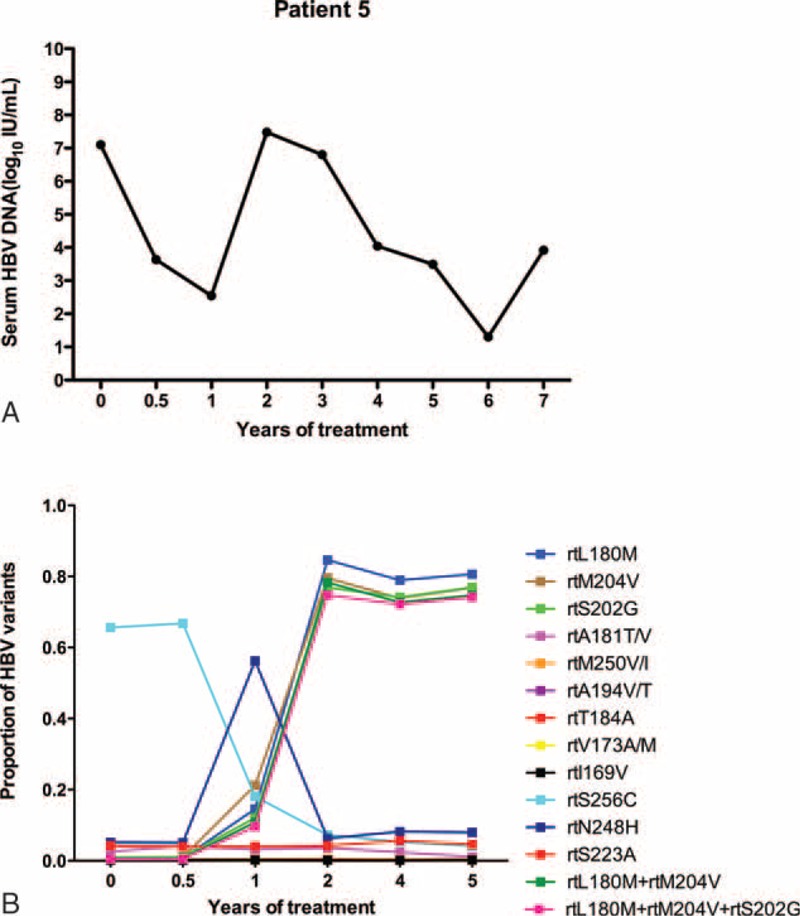
The dynamic changes in HBV DNA levels and resistant variants of the RT domains during ETV treatment of patient 5. (A) The HBV DNA declined to a low level (10^2^ IU/mL) at 1 year, but viral replication increased (10^7^ IU/mL) by 2 years. During gradual ETV therapy, the DNA level began to decrease after a nadir at 6 years, and a virological breakthrough appeared in the 7th year (10^3^ IU/mL). (B) From 1 to 2 years, the resistant variants (rtL180 M, rtM204 V, rtS202G) began to rise to a high peak (84.60%, 79.56%, 76.88%), and double amino acid substitutions (rtL180M+rtM204 V) and triple substitutions (rtL180M+rtM204V+rtS202G) also began to rise. All of the single- and double-resistant variants maintained high values. ETV = entecavir, DNA = di-ribonucleic acid, HBV = hepatitis B virus, RT = reverse transcriptase.

Figure 5A shows that the HBV DNA in patient 5 declined to a low level (10^2^ IU/mL) at 1 year, but that viral replication increased (10^7^ IU/mL) by 2 years. During gradual therapy, the DNA level began to decrease after a nadir at 6 years, and virological breakthrough appeared in the 7th year (10^3^ IU/mL). The results in Figure 5B demonstrated that from 1 to 2 years, resistant variants (rtL180 M, rtM204 V, rtS202G) began to rise to a high peak (84.60%, 79.56%, 76.88%) and that double amino acid substitutions (rtL180M+rtM204 V) and triple substitutions (rtL180M+rtM204V+rtS202G) also began to rise. All of the single- and double-resistant variants maintained high values.

## DISCUSSION

In this study, UDPS was used to analyze the relationships between NAr substitutions and curative antiviral effects in 32 treatment-naive patients (PVRs = 20, CVRs = 12). In our cohort, the rate of PVR (42.6%, 26/61) was a little higher than Zoutendijk R et al reported (21%).^20^ The level of DNA is relatively higher at baseline, and the major genotypes were B and C. The plausible reasons for the differences were the HBV DNA level, genotype, and host factors. We further analyzed the dynamic changes in QS in 5 PVRs undergoing long-term therapy.

Since the concept of QS was introduced in HCV research, studies have reported that hepatitis C QS at baseline are related to the early virological response during interferon and ribavirin treatment.^26–28^ In recent decades, certain reports have specifically investigate HBV QS complexity and diversity. HBV QS evolution in the early stage of NA therapy can be used to predict the long-term virological response.^15,29^ Most researchers use cloning to assess QS evolution; however, this method's precision and sensitivity must be improved. UDPS analysis can detect a lower frequency (<1%) of resistant variants.^30^ In our study, QS complexities and diversities were not significantly different between 2 groups at baseline. The results support the idea that hepatitis B QS cannot affect curative effects before therapy, which is different from what has been observed in chronic HCV.

UDPS indicated that NAr mutations were pre-existent at low percentages (ranging from 0.1% to 6.7%) at baseline, including rtV173A/M, rtL180 M, rtA181 V/T, rtS202G, rtM204I/V, rtV214A, rtQ215R/H, and rtN236T mutations. One study reported that the most commonly detected mutations were M204 V/I, M250 V/I, A181T/V, and N236T.^31^ It was similar with the results of Nishijima et al, the pre-existing low-abundant mutations did exist at baseline. In contrast, 1 study reported that these resistance mutations were not identified in 16 treatment-naive patients.^32^ A limited amount of information is currently available regarding pre-existing mutations, so whether the frequencies of these mutations are associated with populations or genotypes remains to be further verified. In the present study, each drug-resistant variant at baseline was comparable between the 2 groups. Another study reported that pre-existent resistance mutations at a low frequency cannot be used to predict the virological response to NA therapy.^33^ In the present study, 7 patients harbored the rtL180 M, rtS202G, and rtM204I/V substitutions at a frequency of >1% at baseline, 5 of these patients belonged to the PVR group, and the other 2 belonged to the CVR group. Although significant differences were not observed between these groups, a high proportion of patients experienced ETV-related mutations in the PVR group; in the future, it will be important to expand the sample size to achieve statistical significance. The next follow-up revealed that the substitutions had decreased to <1% in 5 of the 6 patients.

The goal of therapy is to suppress viral replication to avoid disease progression.^34–36^ Viral responses can be used to predict long-term outcomes and effectively suppress viral replication.^8,17,18^ Guidelines suggest that patients should change or combine drugs when treatment based on less potent drugs fails.^8,18,37^ There are few data regarding ETV in particular, which, as the most potent antiviral drug, is widely used in the clinic. Studies have demonstrated that primary nonresponders may experience a virological response while receiving long-term ETV monotherapy (for 3 years), which provides strong evidence for reappraisal of the current guidelines.^20^ Long-term persistence of the virus can influence hepatocyte damage and cause drug-resistance mutations, which can subsequently result in virological breakthrough. These conclusions have been confirmed using less potent drugs, which pose a higher risk of antiviral resistance.^38–40^ Few data are available regarding the dynamics of HBV QS and genotypic mutations after long-term treatment with ETV. Certain articles have reported that genotypic resistance to ETV was not detected in a PVR group at week 48.^20^ Moreover, few data have been obtained regarding predicting the risks of resistance mutations during long-term ETV therapy. In the present study, using UDPS, the dynamic changes in HBV QS and genotypic mutations were explored under the pressure of ETV treatment in 5 patients. At baseline, patient 1 harbored rtL180 M (2.09%), rtS202G (3.42%), and rtM204 V (3.45%) mutations, the frequencies of which rose to 19.12%, 18.33%, and 19.95%, respectively, after 1 year of treatment, but these changes did not cause virological breakthrough. These resistance mutations decreased to <1% by 2 years. Patients 1 through 4 demonstrated that HBV variants resistant to NAs undergo no major changes (<20%) during ETV treatment, and these patients ultimately achieved a virological response. In patient 5, when the resistant variants (rtL180 M, rtM204 V, rtS202G) began to rise to a high peak (84.60%, 79.56%, 76.88%) at 2 years, virological breakthrough occurred. This result indicates that there are genotypic mutations of concern during long-term therapy, and clinicians must detect the changes in resistant variants to prevent virological breakthrough.

In conclusion, NAr substitutions are found at frequencies of 0.10% to 6.7% before therapy as pre-existing substitutions. Genotypic resistances to ETV were detected in the partial responders with long-term therapy. However, a large sample will be necessary to explore the threshold of frequency of resistance mutations to determine whether to adjust the therapy programs. In addition to known NAr mutations, several novel mutations were identified, including rtN248H, rtS223A, rtS256C, and rtI224 V; whether these mutations are associated with curative effects remains to be further verified as well.
